# Therapeutic Response of Soft Tissue Sarcoma With Novel SS18-POU5F1 Fusion: A Case Report

**DOI:** 10.3389/fonc.2021.666946

**Published:** 2021-06-24

**Authors:** Zengjun Liu, Hongtu Yuan, Mingyong Han

**Affiliations:** ^1^ Tumor Research and Therapy Center, Shandong Provincial Hospital, Cheeloo College of Medicine, Shandong University, Jinan, China; ^2^ Rare Tumors Department, Shandong Cancer Hospital and Institute, Shandong First Medical University and Shandong Academy of Medical Sciences, Jinan, China; ^3^ Department of Pathology, Shandong Cancer Hospital and Institute, Shandong First Medical University and Shandong Academy of Medical Sciences, Jinan, China

**Keywords:** soft tissue sarcoma, SS18, POU5F1, fusion, therapeutic response

## Abstract

A novel SS18-POU5F1 fusion gene was recently reported in soft tissue sarcoma occurring in three adolescent and young adult patients. Herein, we firstly reported the treatment response of SS18-POU5F1 sarcoma to immune checkpoint inhibitor, angiogenesis inhibitor, chemotherapy and radiotherapy. Our patient demonstrated no response to various systemic therapies including immune checkpoint inhibitor, angiogenesis inhibitor and chemotherapy. However, we noted that the SS18-POU5F1 sarcoma had a quick, robust but transient clinical response to radiotherapy. Further studies are needed to elucidate the mechanism underlying the different tumor response to radiotherapy and systemic therapy in this kind of tumor.

## Introduction

Recent advances in clinical molecular diagnostics have provided greater understanding of soft tissue sarcoma. Herein, we report the clinical course of a sarcoma patient with a novel SS18-POU5F1 fusion gene.

## Case Presentation

An 18-year-old female presented to the local hospital with a painless immovable non-tender 5×5×2 cm cystic-solid mixed mass founded in the soft tissue of the left groin that had been present for 2 months. The mass was widely excised with negative margins. Microscopically, the tumor was composed of two kinds of cells: the undifferentiated small round cells and the epithelioid big cells. The cells arranged in solid sheets and trabeculae. The tumor had sheet necrosis and large thick wall blood vessels, and some areas were rich in blood vessels. The cytoplasm was scant and eosinophilic. The nuclei were small, round and uniform in shape. Nucleoli were generally small but prominent. There was brisk mitotic activity (20 mitoses per 10 high-power fields). Immunohistochemically, an extensive panel revealed expression of E-cadherin, β-catenin, INI-1, and partly expression for panCK, EMA, CD31, Bcl-2, Vimentin. The immunoreactivity for Fli-1 was weak; the expression for CD99 and S-100 was focal. The tumor was negative for TFE3, CD34, HMB45, MyoD1, Myogenin, F8, ERG. The ki67 proliferative index was approximately 60%. The targeted DNA and RNA sequencing of a panel of 1084 genes revealed a novel SS18-POU5F1 fusion. The transcript involved SS18 exon 9, and POU5F1 exon 2. The tumor mutation burden is 0.47 mutations per megabase. Immunohistochemical analysis of programmed death-ligand 1 expression using the murine 22C-3 antibody revealed a tumor proportion score 3%. The microsatellite stability analysis showed that the tumor was microsatellite stable.

**Figure 1:**
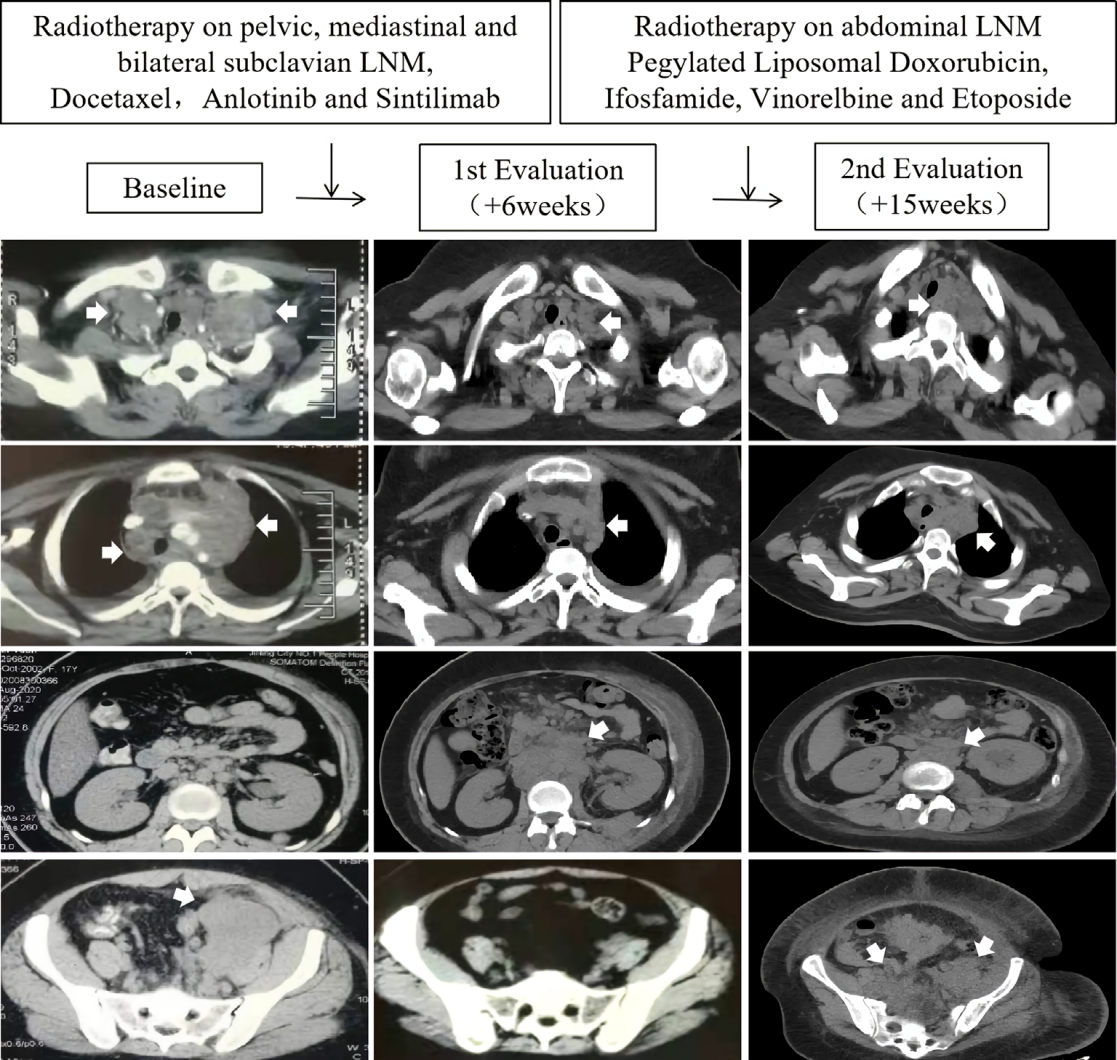
Scans at baseline, first evaluation (+6 weeks), and second evaluation (+15 weeks) in a SS18-POU5F1 sarcoma patient with multiple lymph node metastases (LNM) who accepted immune checkpoint inhibitor, angiogenesis inhibitor, chemotherapy and radiotherapy.

Four weeks later, the patient presented to the local hospital again with progressively worsening edema of the left lower limb. A contrast-enhanced total-body computed tomography (CT) scan (head to foot) revealed pelvic, mediastinal and bilateral subclavian lymphadenopathy (**Figure 1**, Baseline). Ultrasound-guided biopsy of the subclavian lymphadenopathy confirmed similar pathological findings to the mass in the left thigh. On further staging, no other distant metastasis was detected.

She accepted 2 cycles of therapy with docetaxel, anlotinib (a multitargeted angiogenesis inhibitor) and sintilimab (a humanized programmed death receptor-1 monoclonal antibody). Concurrently, she accepted 39 Gy (3Gy×13 fractions) radiotherapy on pelvic, mediastinal and bilateral subclavian lymph node metastases. The edema of the left lower limb gradually disappear during the radiotherapy. A partial response was observed in the lesions within the radiation fields. However, unfortunately, a progressive disease was confirmed in the abdominal regions outside the radiation fields (**Figure 1**, 1st evaluation). The patient complained of progressively worsening back pain. Sitting up and leaning forward tended to ease the pain, while lying down worsen it.

She was referred to our rare tumors clinic for radiotherapy on the abdominal lymph node metastases (2.5 Gy ×15 fractions). Concurrently, she accepted chemotherapy with pegylated liposomal doxorubicin, ifosfamide, vinorelbine and etoposide within two months. The abdominal lesions shrank significantly during radiotherapy. The back pain resolved. However, unfortunately, severe edema of both lower limbs and inferior abdominal wall relapsed during the late stage of abdominal radiotherapy. A total-body CT scan (head to foot) showed progression of the left inguinal, pelvic, mediastinal and bilateral subclavian lymph node metastases (**Figure 1**, 2nd evaluation). She died of systemic metastases 8 months after the diagnosis.

## Discussion

A novel SS18-POU5F1 fusion gene was recently described as case reports of soft tissue sarcoma occurring in three adolescent and young adult patients (1, 2). All previous and present cases have relatively similar histopathologic features. All tumors were composed of sheets and trabeculae of round to epithelioid cells with scant cytoplasm and round uniform nuclei, exhibiting a high mitotic activity and necrosis. Immunohistochemically, all tumors variably expressed cytokeratin, EMA and S-100, suggesting the possibility of myoepithelial carcinoma, or perhaps a poorly differentiated synovial sarcoma. Genomic analysis revealed a novel SS18-POU5F1 fusion mapping to exon 10 of SS18 and exon 2 of POU5F1 in previous reports, and exon 9 of SS18 and exon 2 of POU5F1 in our case respectively. RNA sequencing analysis of one previous case showed that the SS18-POU5F1 tumors cluster with EWSR1/FUS-POU5F1-positive myoepithelial tumors, suggesting molecular kinship with this group of tumors (1).

In the present case, we firstly reported the treatment response of SS18-POU5F1 sarcoma to immune checkpoint inhibitor, angiogenesis inhibitor, chemotherapy and radiotherapy. Our patient demonstrated no response to various systemic therapies including immune checkpoint inhibitor, angiogenesis inhibitor and chemotherapy. However, we noted that the SS18-POU5F1 sarcoma had a quick, robust but transient clinical response to radiotherapy. One possible explanation for the transient response may be the specifically designed low-dose radiation protocol in order to avoid injury to adjacent radiation vulnerable tissues. Although the response was transient, we propose that the fast and favorable response to radiotherapy may play a vital role in the neoadjuvant setting.

Radioactive seeds implantation may be an alternative local salvage modality to provide a long-standing exposure to a mild dose of radiation.

## Conclusion

We firstly reported the different treatment response of SS18-POU5F1 sarcoma to immune checkpoint inhibitor, angiogenesis inhibitor, chemotherapy and radiotherapy. We recognize that further studies are needed to elucidate the mechanism underlying the different tumor response to radiotherapy and systemic therapy in this kind of tumor.

## Data Availability Statement

The original contributions presented in the study are included in the article/supplementary material. Further inquiries can be directed to the corresponding author.

## Ethics Statement

The studies involving human participants were reviewed and approved by Medical Ethics Committee in Shandong Cancer Hospital and Institute. The patients/participants provided their written informed consent to participate in this study. Written informed consent was obtained from the individual(s) for the publication of any potentially identifiable images or data included in this article.

## Author Contributions

ZL, HY, and MH performed image acquisition and completed the manuscript. All authors contributed to the article and approved the submitted version.

## Conflict of Interest

The authors declare that the research was conducted in the absence of any commercial or financial relationships that could be construed as a potential conflict of interest.

## References

[1] Antonescu CR Agaram NP Sung YS Zhang L Dickson BC. Undifferentiated Round Cell Sarcomas With Novel SS18-POU5F1 Fusions. Genes Chromosomes Cancer (2020) 59(11):620–6. 10.1002/gcc.22879 PMC8115304 32557980

[2] Shenoy A Newsom K Gray B Zhang Y Lagmay JP Islam S. Malignant Round Cell Tumor With SS18-POU5F1 Fusion: Is it a Myoepithelial Neoplasm, a Synovial Sarcoma or a New Entity? Histopathology (2020) 77(4):681–4. 10.1111/his.14171 32516451

